# Concordance of the Resting State Networks in Typically Developing, 6-to 7-Year-Old Children and Healthy Adults

**DOI:** 10.3389/fnhum.2017.00199

**Published:** 2017-04-25

**Authors:** Cody L. Thornburgh, Shalini Narayana, Roozbeh Rezaie, Bella N. Bydlinski, Frances A. Tylavsky, Andrew C. Papanicolaou, Asim F. Choudhri, Eszter Völgyi

**Affiliations:** ^1^Division of Clinical Neurosciences, Department of Pediatrics, University of Tennessee Health Science CenterMemphis, TN, USA; ^2^College of Medicine, University of Tennessee Health Science CenterMemphis, TN, USA; ^3^Neuroscience Institute, Le Bonheur Children's HospitalMemphis, TN, USA; ^4^Department of Anatomy and Neurobiology, University of Tennessee Health Science CenterMemphis, TN, USA; ^5^Department of Preventive Medicine, University of Tennessee Health Science CenterMemphis, TN, USA; ^6^Department of Radiology, University of Tennessee Health Science CenterMemphis, TN, USA; ^7^Department of Neurosurgery, University of Tennessee Health Science CenterMemphis, TN, USA; ^8^Department of Family and Community Medicine, Health Disparities Research Center of Excellence, Meharry Medical CollegeNashville, TN, USA

**Keywords:** resting fMRI, children, normative, independent component analysis, resting state network

## Abstract

Though fairly well-studied in adults, less is known about the manifestation of resting state networks (RSN) in children. We examined the validity of RSN derived in an ethnically diverse group of typically developing 6- to 7-year-old children. We hypothesized that the RSNs in young children would be robust and would reliably show significant concordance with previously published RSN in adults. Additionally, we hypothesized that a smaller sample size using this robust technique would be comparable in quality to pediatric RSNs found in a larger cohort study. Furthermore, we posited that compared to the adult RSNs, the primary sensorimotor and the default mode networks (DMNs) in this pediatric group would demonstrate the greatest correspondence, while the executive function networks would exhibit a lesser degree of spatial overlap. Resting state functional magnetic resonance images (rs-fMRI) were acquired in 18 children between 6 and 7 years recruited from an ethnically diverse population in the Mid-South region of the United States. Twenty RSNs were derived using group independent component analysis and their spatial correspondence with previously published adult RSNs was examined. We demonstrate that the rs-fMRI in this group can be deconstructed into the fundamental RSN as all the major RSNs previously described in adults and in a large sample that included older children can be observed in our sample of young children. Further, the primary visual, auditory, and somatosensory networks, as well as the default mode, and frontoparietal networks derived in this group exhibited a greater spatial concordance with those seen in adults. The motor, temporoparietal, executive control, dorsal attention, and cerebellar networks in children had less spatial overlap with the corresponding RSNs in adults. Our findings suggest that several salient RSNs can be mapped reliably in small and diverse pediatric cohort within a narrow age range and the evolution of these RSNs can be studied reliably in such groups during early childhood and adolescence.

## Introduction

Over the course of the past decade, there has been an increased effort toward understanding the functional architecture of brain, specifically in relation to resting brain networks (Gusnard et al., 2001). The most common approach for characterizing the resting brain networks is to quantify the temporal correlation of neuronal activity between anatomically distant brain regions, termed functional connectivity. Most commonly, the distributed functional connectivity patterns between brain areas that share similar variation in their activity over time are identified using a data driven technique, the independent component analysis (ICA) (Jafri et al., 2008; Sohn et al., 2012). Each independent component represents a functional network that consists of constituent brain regions having a closely correlated time course. Several such components or networks, each with its own temporal characteristic can be simultaneously derived without specifying brain regions. Utilizing the ICA approach, large-scale fMRI studies of neurologically intact adults have identified a set of robust and reproducible brain networks, generally acknowledged to reflect the normative profile of brain activity during rest (Smith et al., 2009; Zuo et al., 2010; Doucet et al., 2011; Yeo et al., 2011; Allen et al., 2014). The functional relevance of these resting state networks (RSN) are interpreted, primarily, on the basis of their spatial profile and include: default mode (DMN), medial, lateral, and parietal visual, auditory, somatosensory, motor, attention, executive control, cerebellar, and frontoparietal networks.

Though fairly well-studied in adults, less is known about the manifestation of these functional networks in typically developing children. In the few published studies to date, derivation of RSNs using fMRI in typically developing children has been attempted in a small number of infants and toddlers during various stages of sleep (Fransson et al., 2011; Manning et al., 2013), in children under sedation (Funakoshi et al., 2016), in awake children (5–10 years old; de Bie et al., 2012), and adolescents (9–15 years old; Littow et al., 2010; Jolles et al., 2011; Thomason et al., 2011). In a small study on six healthy preschool children aged 2–5 years during stage 3, non-rapid eye movement sleep, 18 non-artifactual RSNs were derived (Manning et al., 2013). While the sensorimotor, auditory, visual, cerebellar, and executive control networks were identifiable, the RSNs were contaminated by artifacts since noise components from individual data were not removed. In another study of 18 infants (average age 10 months), most connected regions in these children were limited to primary motor and sensory cortices, where as in adults, prefrontal and association areas were better connected (Fransson et al., 2011). The DMN was seen to be present in children (1–8 years of age) even under sedation, albeit with decreased connectivity than that noted in adults (Funakoshi et al., 2016). However, this study included mainly boys (14/15) and did not examine other RSNs. The RSNs supporting basic motor and sensory functions were found to have a functional organization similar to mature adult patterns in a study on eighteen 5- to 8-year-old children in an awake state. However, in these children, the DMN and RSNs relating to attention and executive control were observed to be more fragmented than the corresponding adult networks (de Bie et al., 2012). Recently, a large sample study in Dutch children (6–10 years old) extracted RSNs from 536 children (Muetzel et al., 2016). Similar to previous studies in awake children, the large cohort study found cerebellar, default-mode, executive control, frontoparietal, parietal, sensorimotor, and visual networks to be highly reproducible. Overall, the studies of RSNs in awake children, the ICA-derived primary networks like visual, auditory, somatosensory, and motor networks closely resemble adult networks in their spatial configuration, while the networks that mediate higher cognition including executive control, and frontoparietal networks demonstrate greater variability with fewer regions in the putative RSNs in children (de Bie et al., 2012; Muetzel et al., 2016).

While the studies in young children provide a good foundation to future studies of RSNs in children in this age, they do have a few disadvantages: 1. There is variability in the number of derived networks that range from 20 to 70, which precludes direct comparisons (Smith et al., 2009; de Bie et al., 2012; Muetzel et al., 2016); 2. The age ranges of 6–10 years (Muetzel et al., 2016) and 5–8 years (de Bie et al., 2012) are large, especially since there is significant motor and cognitive development during this time span; In fact, age related changes in default mode and executive control networks are observed in a cohort of 6–10 year olds (Muetzel et al., 2016); 3. Often, the studies examine few selected RSNs. 4. Racially and socioeconomically diverse populations have not been widely studied as most studies are from European countries with mostly Caucasian children from higher socioeconomic strata (Littow et al., 2010; de Bie et al., 2012; Muetzel et al., 2016). Hence, this study aims to address the lack of diversity in current normative developmental studies by studying a more inclusive population and a narrower age range of 6–7 year old children in order to identify age specific RSNs.

Accordingly, the purpose of this study was to derive resting-state networks in a diverse group of 6- to 7-year-old children from the Mid-South region of the United States and compare their spatial pattern with established RSNs in adults and approximately age-matched children. We quantified the spatial concordance between the RSNs identified in our study population and the publicly available 20 Component adult sample set generated by Smith et al. (2009). However, as the RSNs in children between 5 and 10 years (de Bie et al., 2012; Muetzel et al., 2016) were not publically available, comparison between our study and the previous pediatric studies was limited to visual evaluation. We predicted that the RSNs in young children would be robust and could be mapped reliably in our diverse cohort. We also expected that the RSNs of typically developing 6- to 7-year-old children would be qualitatively similar to previously reported pediatric RSNs and show significant spatial overlap with published adult RSNs. Consistent with previous reports, we expected that primary sensory networks, i.e., visual, auditory, and somatosensory as well as primary motor and the default mode networks (DMNs) in this racially diverse pediatric group would demonstrate significant spatial concordance with those reported in adult cohort studies, while higher cognition networks (like the executive control and frontoparietal networks) would exhibit a lesser degree of spatial overlap.

## Materials and methods

### Participants

Participants were recruited from the Conditions Affecting Neurocognitive Development and Learning in Early Childhood (CANDLE) study (www.candlestudy.com) cohort enrolled between 2007 and 2011. Out of 893 children who had completed their 4-year visit at the time of recruitment for the current study, 492 children fulfilled the age criteria of turning 6 years between January 1st and November 7th of 2015. From this group, 200 children meeting the following criteria were identified as potential participants: 1. No prenatal exposure to drug, alcohol or smoking; 2. Born full term; 3. An average or above score on the full scale Stanford-Binet Intelligence Scales (SB5) (Roid, 2003) at the age of 4; 4. Score in the normal range on the Child Behavior Checklist for Ages 1½–5 (CBCL-3) (Achenbach and Rescorla, 2000; Achenbach and Ruffle, 2000) syndrome scale, DSM-oriented scales, broadband scale, or total problems score; and 5. Children without history of autism spectrum disorder screened by the Modified Checklist for Autism in Toddlers (M-CHAT; Robins et al., 1999, 2001). Flyers were mailed out in batches based on the child's age (older children were invited first). Parents of 70 children contacted the study personnel and expressed their interest in participating in the study. Of them, 20 children having metal in body or mouth, were left handed, had a history of neurological disease, concussion, or head injury were not included in the study. Thirty-seven typically developing children between the ages of 6 and 7 years were enrolled in the study. The cohort of 37 children with a mean age of 6.7 years included 20 girls and 17 boys and their racial profiles (56% African American, and 44% Caucasian) were representative of the area of recruitment. The structural brain MRI scan was reviewed for abnormalities by a Board Certified Pediatric Neuroradiologist. Participants' right handedness was confirmed by the short form of the Edinburgh Handedness Inventory (Veale, 2014) and observation by the research staff. Seven participants were excluded from the study post-consent where 2 were found to be left handed, 1 had prenatal tobacco exposure, 1 had prenatal alcohol exposure, and 3 had scores on CBCL-3 in the clinical range. Two children could not tolerate the fMRI scanning and 10 participants were further excluded from fMRI analysis due to excessive motion artifacts. Therefore, the sample size included in the final analysis was 18 children. Their mean age was 6.7 years, and the gender composition of the children was equal number of males and females. The racial profile of the study group included equal distribution of both African-American and Caucasian children. Other characteristics of the participants are detailed in Table 1.

**Table 1:** **Basic characteristics of study population (*n* = 18)**.

	**Mean ± SD**	**Range**
Age (years)	6.7 ± 0.5	6.3–7.9
Sex (M/F, n)	9/9	
Height (cm)	122.1 ± 5.1	111.0–128.8
Weight (kg)	25.0 ± 4.5	17.2–36.1
BMI percentile	62.3 ± 24.7	15.0–98.7
Race (AA/CA, n)	9/9	
Gestation age (weeks)	39.3	36.5–41.1
Full-scale IQ	102	90–128
Avg. RMS relative motion (mm)	0.17	0.03–0.49

*BMI, Body Mass Index; AA, Non-Hispanic, African American; CA, Non-Hispanic, Caucasian; IQ, intelligence quotient; RMS, root mean square*.

The study was performed in accordance with the Declaration of Helsinki and approved by the Institutional Review Board of the University of Tennessee Health Science Center. The study procedures were explained in an age-appropriate manner to the participants, and a written informed consent was obtained from their legally authorized representatives, and the families were compensated for their time.

### Data acquisition

A Siemens 3T Verio MRI Scanner (Siemens AG, Munich, DE) with a 12-channel head coil was used to perform structural and functional brain imaging. A T2^*^-weighted gradient-echo echo-planar-imaging BOLD-fMRI was acquired as the children lay still in the scanner with closed eyes. Two hundred and three volumes with a voxel size of 2.55 × 2.55 × 3.5 mm, TR of 3,000s, TE of 30s, a flip angle of 90°, and Field of view = 256 × 204 × 140 were acquired in an ascending slice order. After the fMRI, a high-resolution anatomical image was acquired using a T1 weighted 3D sequence (TR/TE/flip angle = 1,900/2.93/9°) with slice-select inversion recovery pulse (TI = 900 ms), field of view = 512 × 512 × 176, and 0.5 × 0.5 × 1 mm spatial resolution. The children practiced lying still with their eyes closed in the MRI scanner prior to scanning. Children were allowed to watch cartoons during the anatomical MRI session. As part of this study, diffusion tensor images were also acquired and will be reported separately.

### Preprocessing and registration

The initial 3 volumes of the fMRI data were discarded and the remaining 200 volumes acquired when fMRI signals were in steady-state were further analyzed. The DICOM images were converted to the NIFTI format using the Multi-image Analysis GUI (Mango; ric.uthscsa.edu/mango/). Structural images were stripped of the cranial and outer visceral layer using the Brain Extraction Tool (BET) plug-in within Mango (Smith, 2002). Both functional and structural images were visually inspected to look for major movement artifacts and usability of data and removed from the study as necessary.

The fMRI data were processed using FMRIB Software Library (FSL) (v5.0; http://fsl.fmrib.ox.ac.uk/fsl/; Jenkinson et al., 2012). The data were preprocessed and corrected for slice timing and motion artifacts using motion correction based on FMRIB's Linear Image Registration Tool (MCFLIRT; Jenkinson et al., 2002). The absolute and relative movements were evaluated and 4D volumes with movement >2 mm were removed. This resulted in exclusion of 10 participants, resulting in a final sample size of 18. Brain extraction was carried out on the fMRI data using BET (Smith, 2002). fMRI images were smoothed (Full width half maximum—FWHM 5 mm), normalized by a single multiplicative factor, and high pass temporal filtering applied at a sigma = 50.0s (Jenkinson and Smith, 2001; Jenkinson et al., 2002). FSL's FMRIB's Linear Image Registration Tool (FLIRT) algorithm registered each participant's fMRI to their respective anatomical MRI using boundary based registration and to the Montreal Neurological Institute (MNI) asymmetric 4.5–8.5 year old standard brain (http://nist.mni.mcgill.ca/; Fonov et al., 2011).

### Individual independent component analysis

The ICAs were decomposed from each preprocessed, 4D volume into using single-session ICA using FSL's Multivariate Exploratory Linear Optimized Decomposition into Independent Components tool (MELODIC v3.14) (Beckmann and Smith, 2004). The time course and the spatial distribution of components in each participant were visually inspected and components identified to be motion or physiological artifacts resulting from large blood vessel and cerebral spinal fluid pulsations were removed using the fsl_regfilt program within the FSL package. In addition, components that had sudden spikes in the time course or significant high frequency content were also rejected. This step was performed with three thresholds: A liberal threshold where 20–25% components were identified as noise and removed, moderate where 40–60% components were identified as noise and removed, and conservative where 60–80% components were identified as noise and removed. In this scenario, with liberal threshold, any noisy IC that had some brain-derived components was not removed. However, with conservative threshold, such ICs were excluded. The resulting filtered and de-noised 4D volumes were transformed to standard space and input into the group analysis pipeline.

### Group independent component analysis

The cleaned and transformed volumes (*n* = 18) were run through the multisession temporal concatenation option in MELODIC to generate group level independent component networks (Beckmann and Smith, 2005). We restricted the dimensionality to 20 components as the study in adults that we are comparing against also used similar values. The similarity of spatial distribution between the RSN components derived from this group analysis and the 20 Component adult sample set, as generated by Smith et al. (2009) was examined using Pearson cross-correlation algorithm implemented in FSL (fslcc). We applied Fisher's r-to-z transform using a conservative degrees-of-freedom value of 500 (number of independent resolution elements) and converted the resultant *z* score to a *P*-value (Smith et al., 2009). Using this method, we found that comparisons with Pearson's *r* > 0.204 were significantly spatially correlated (*P* < 0.0001). The RSNs identified in our cohort were also compared to previously published pediatric RSNs (de Bie et al., 2012; Muetzel et al., 2016) by visual inspection.

### Dual regression analysis

In order to investigate if any of the demographic, anthropometric, and cognitive metrics influenced the spatial composition of each participant's RSNs, we performed a dual regression analysis. The demographic variables examined included gender, race, age, and gestational age at birth. The anthropometric parameters including the raw value and their gender and age adjusted percentiles of height, weight, and body mass index (BMI), and the cognitive variable of full-scale intellectual quotient (IQ) were also used as regressors. First, the degree to which each group level RSN was represented in each subject was estimated. For this, the spatial map of each group level RSN was regressed into each subject's 4D volume and subject specific time courses were generated and these time courses were regressed into the same 4D volume to derive a subject specific set of RSNs. Then, the associations between each variable listed above and subject specific RSNs were examined using FSL's Randomize tool (Winkler et al., 2014) with 5,000 permutations and corrected for multiple comparisons using threshold free cluster enhancement threshold.

## Results

### Individual independent component analysis

On average, 48 ± 6 ICs were derived in each participant. Using a liberal threshold, 12.5 ± 4.0 components were identified as noise and removed. The number of noise components removed was 25.2 ± 4.3 with a moderate threshold, and 32.1 ± 7.7 components were identified as noise and removed with a conservative threshold. Of the three noise removal thresholds examined, the moderate noise threshold that removed 40–60% of components as artifacts, was found to be optimal and was applied to the data.

### Group independent component analysis

**Figure 1:**
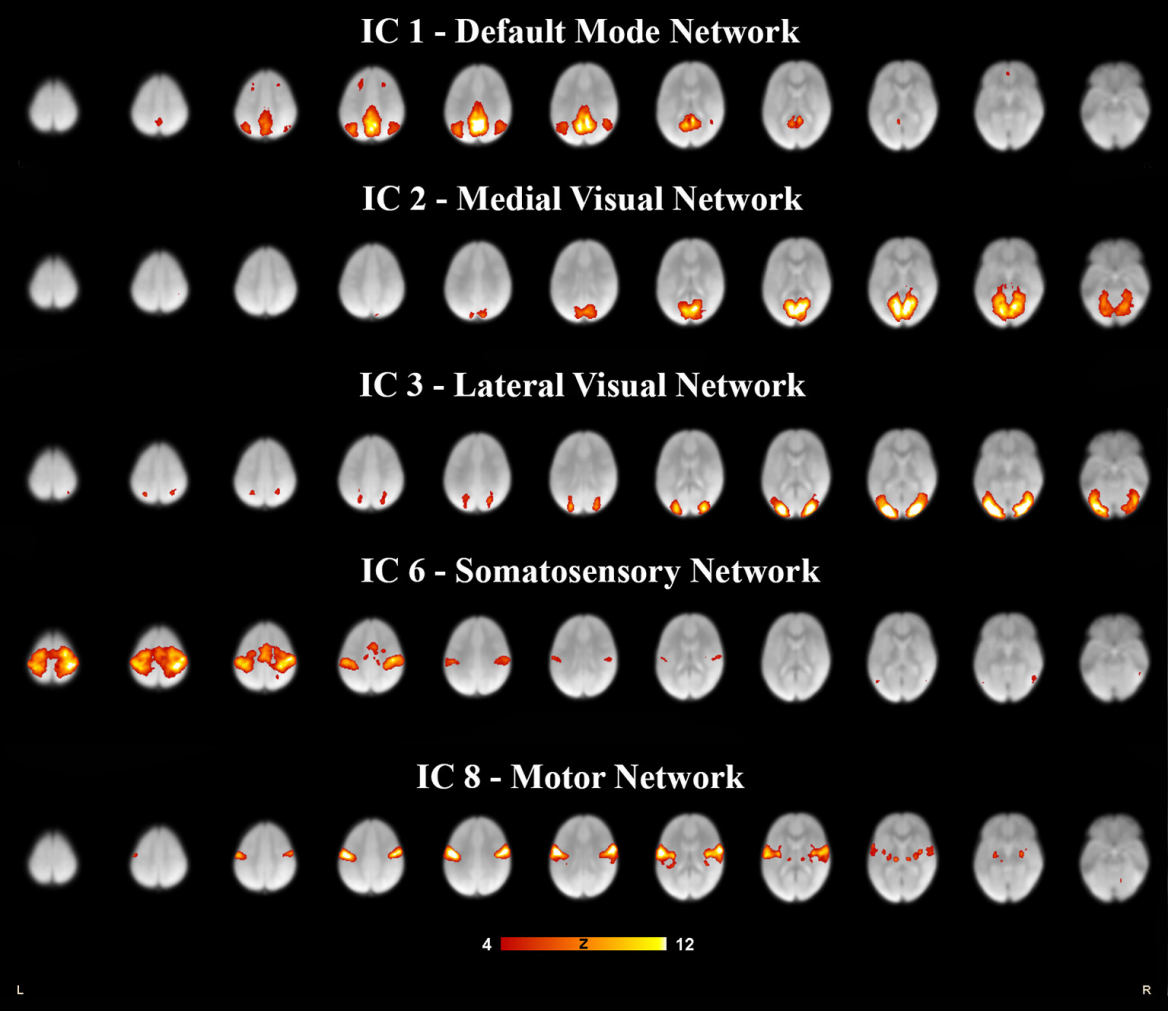
**Five resting state networks derived from 6- to 7- year olds rs-fMRI selected for relevance and high correlation**. Each independent component was paired to the components derived in adults (Smith et al., 2009), for spatial overlap which was quantified as a spatial cross correlation coefficients. The networks with a spatial cross-correlation coefficient (r) ≥ 0.204 relating to *P* < 0.0001 are depicted here. The images are shown in neurological convention, with the left side of the brain is represented in the left side of the figure. All overlays were made from the z-statistical images and thresholded to 4 < Z < 12.

**Figure 2:**
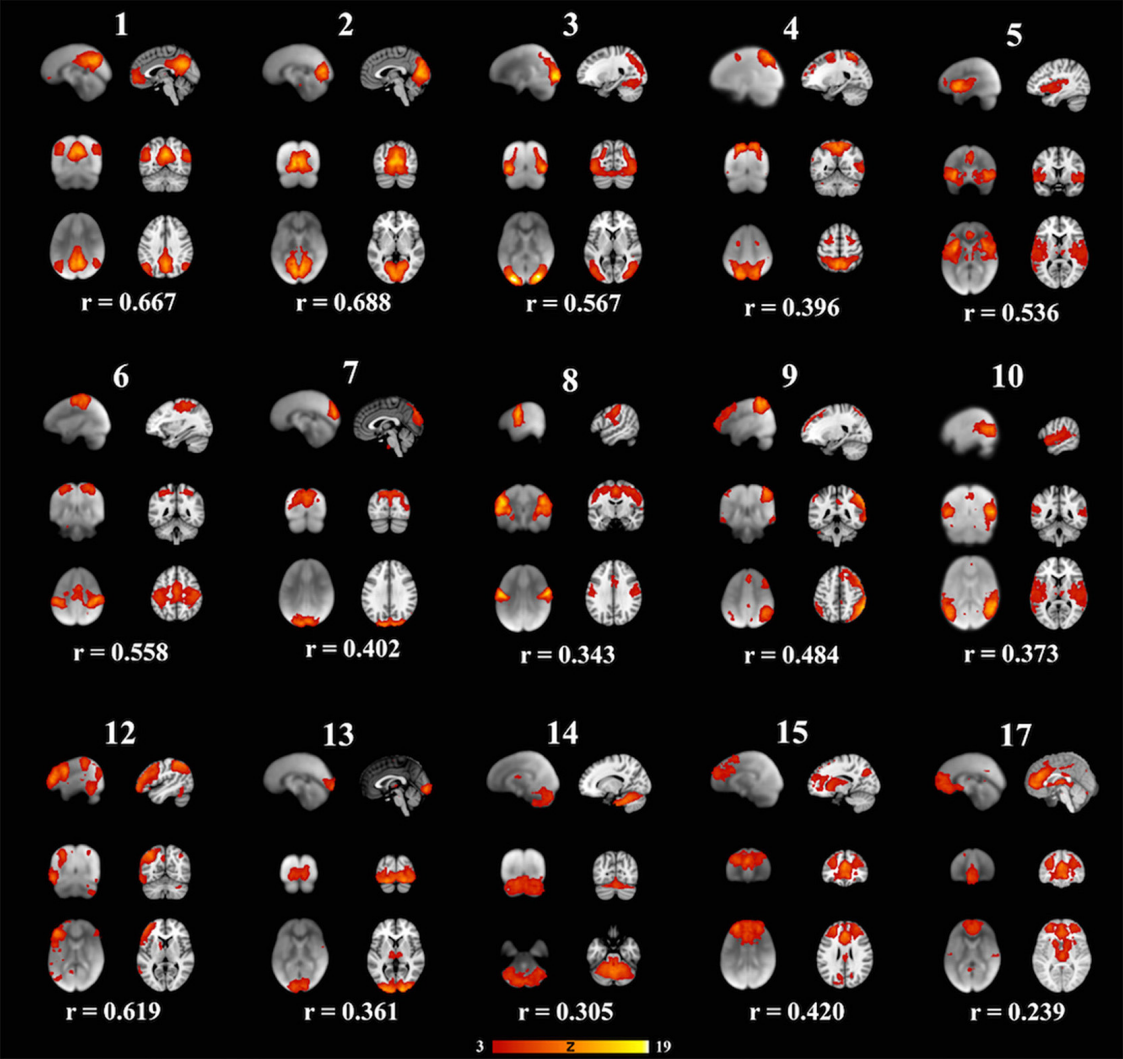
**Fifteen well-matched resting state networks from the 20-dimensional component analysis to the 20-dimensional study of a healthy adult population, as described elsewhere (Smith et al., 2009)**. Each individual component is shown with three informative orthogonal slices with the corresponding adult network slices at the given MNI152 standard space coordinates. The left column represents resting state networks in 6-7-year-old children overlaid on the group's averaged anatomical brain. The right column represents the closest adult network overlaid on the adult MNI brain atlas. The networks demonstrated spatial cross-correlation coefficient (r) > 0.204, which based off methods outlined in Smith et al. (2009) relates to *P* < 0.0001. All overlays were made from the z-statistical images and thresholded to 3 < Z < 19. The coronal and axial images are shown in neurological convention, with the left side of the brain is represented in the left side of the figure.

Each subject contributed 200, three second volumes to the group analysis for a total of 3,600 3D volumes. At a dimensionality of 20, 15 RSNs were found to be brain-derived networks consistent with previously described networks in adults and children. Five of the remaining RSNs were identified to “noise” resulting from motion, large blood vessel, and cerebral spinal fluid pulsations. The brain-derived RSNs were named in accordance of their spatial distribution and are listed in Table 2. The constituent brain regions in each RSN are also listed in Table 2. The main RSNs identified in our study include the DMN, visual networks—medial, lateral, and occipital pole components, auditory, somatosensory, and motor networks (See Figures 1, 2 and Table 2). Other RSNs identified included the left and right frontoparietal, temporoparietal, executive control, dorsal attention, anterior DMN, and cerebellar networks (see Figure 2).

**Table 2:** **Observed Resting State Networks in 6- to 7- year old typically developing children**.

**RSN Thornburgh 2017**	**Explained variance**	**Total variance**	**RSN Smith 2009**	Cross correlation^*^ (r)	**RSN name**	**Constituent brain regions**
1	6.33	2.58	7	0.667	Default mode network	Bilateral precuneus, posterior cingulate, angular gyrus, anterior cingulate, L-middle frontal gyrus, R-superior frontal gyrus
2	5.95	2.42	6	0.688	Medial visual network	Bilateral cuneus, lingual gyrus
3	5.66	2.3	10	0.567	Lateral visual network	Bilateral middle occipital gyrus, inferior occipital gyrus, precuneus
4	5.52	2.24	15	0.396	Dorsal attention network	Bilateral superior parietal lobule, precuneus, cuneus, middle occipital gyrus, middle frontal gyrus
5	5.39	2.19	3	0.536	Auditory network	Bilateral superior temporal gyrus, transverse temporal gyrus, insula, inferior parietal lobule, inferior frontal gyrus, anterior cingulate
6	5.31	2.16	2	0.558	Somatosensory network	Bilateral supplementary motor area, post central gyrus, precentral gyrus, inferior parietal lobule, middle temporal gyrus, cerebellum
7	5.24	2.13	18	0.402	Upper medial visual network	Bilateral precuneus, cuneus
8	5.08	2.07	2	0.343	Motor network	Bilateral precentral gyrus, post central gyrus, insula, lentiform nucleus, thalamus, cerebellum
9	5.07	2.06	13	0.484	Right frontoparietal network	Superior parietal lobule R > L, inferior parietal lobule R > L, R-middle frontal gyrus, middle temporal gyrus R > L, R-medial frontal gyrus, R-cingulate gyrus
10	5.04	2.05	3	0.373	Temporoparietal network	Bilateral supramarginal gyrus, superior temporal gyrus, middle temporal gyrus, inferior temporal gyrus
12	4.95	2.01	12	0.619	Left frontoparietal network	Left-sided inferior parietal lobule, precuneus, middle frontal gyrus, inferior frontal gyrus, middle temporal gyrus, inferior temporal gyrus
13	4.87	1.96	16	0.361	Occipital pole visual network	Bilateral cuneus, lingual gyrus
14	4.57	1.86	9	0.305	Cerebellar network	Bilateral declive, uvula, pyramis, tuber, inferior semilunar lobule, cerebellar tonsils
15	4.55	1.85	8	0.42	Executive control network	Bilateral medial frontal gyrus, superior frontal gyrus, middle frontal gyrus, anterior cingulate gyrus
17	4.44	1.81	7	0.275	Anterior default mode network	Bilateral medial frontal gyrus, anterior cingulate

Asterisk (^*^) all correlation p < 0.0001. For each of the non-artifactual RSN in the 20 dimensional analyses, the explained and total variance for the study is given. The spatial cross-correlation matched components to the Smith et al. (2009) adult 20 RSNs and the r-values are listed. The RSNs in our study that were significantly correlating to the same adult network are underlined. L, left hemisphere; R, right hemisphere.

We also examined how the RSNs identified in our cohort compared to the previously published adult RSNs in their spatial distribution pattern. The 15 labeled RSNs were compared to the adult cohort data reported by Smith et al. (2009). All 15 of these networks had cross correlation coefficients >0.204, corresponding to *p* < 0.0001 are listed in Table 2 and illustrated in Figure 2. The DMN and the medial visual network demonstrated the highest correlations of the analysis (Figure 2 and Table 2). Of the ten well-matched RSNs (DMN, medial and lateral visual networks, auditory network, frontoparietal networks, and executive control) described by Smith et al. (2009), all were found within our study in 6- to 7-year-old children. A notable difference between the 10 well-matched networks is that in the 6- to 7-year olds the motor (IC 6) and sensory (IC 8) networks were found separated in the children, but in adults, they were described in one single network. The DMN, the sensory networks (visual, auditory, and somatosensory) and the frontoparietal network were found to have the highest spatial cross correlation to the adult RSNs. The motor, temporoparietal, executive control, dorsal attention, and cerebellar networks were found to have a lower level of spatial concordance, as compared to those RSNs described in adults. Lastly, the RSNs that were only partially resolved when compared to adults had the least degree of spatial concordance. For example, the anterior DMN (IC 17) including only the ventromedial frontal brain areas was found to have a spatial cross correlation value of 0.275 when compared to the adult DMN.

Compared to a larger cohort study of similar developmental status that estimated 25 components (Muetzel et al., 2016), only visual inspection between the studies was performed. The findings of our networks appeared to have close resemblance to the large European study and the RSNs reported in this study including the DMN, visual, motor, sensory, executive control, dorsal attention, frontalparietal, and cerebellum networks were clearly demonstrated in the present study of 18 children who were 6 and 7 years old. One exception we noted was that the large study did not report a separately identified auditory RSN, and upon review, the IC 20 listed as insular network most likely includes the auditory network. In the networks related to higher cognitive functions, like the frontoparietal and executive control RSNs, the larger cohort study could resolve several sub-networks (6 out of 25) while our study identified fewer RSNs (2 out of 20).

In another study with similar number and aged cohorts (de Bie et al., 2012) 28 components were estimated at the group level, 15 of which were identified as functionally relevant RSNs. Of these, similar to our findings, the DMN, medial and lateral visual, motor, somatosensory, auditory, and left frontoparietal networks were clearly identifiable. However, similar to the large pediatric cohort study, RSNs that were identified as one network in the present study were found to be distributed across 2–3 RSNs in the study by de Bie et al. (2012). For instance, the RSN labeled auditory network in our study included temporal regions and cingulate cortex, while in the de Bie study (de Bie et al., 2012) reported two separate networks, one with only temporal regions and another with the cingulate cortex. This finding is most likely a result of decomposing the data into fewer components in our study.

### Dual regression analysis

No correlations of statistical significance were found for any of the parameters that we examined as a regressor. The negative findings of these analyses is expected given the cohort consisted of healthy, typically developing children tightly controlled for age and IQ (see Table 1).

## Discussion

We have shown that in a small sample of racially diverse, healthy, typically developing, right handed, 6- to 7-year-old children, resting-state fMRI data can be deconstructed into the fundamental RSN shown in normal adult populations through an ICA. All the major RSNs previously described in adults (Smith et al., 2009) and in a large sample that included older children (Muetzel et al., 2016) were observed in our smaller sample of young children. Our findings were also consistent with RSNs reported in a study with similar number of 5-to 8- year old children (de Bie et al., 2012). To the best of our knowledge, this is the first demonstration of RSNs in the awake state in a group consisting of typically developing children in a narrow age range of 6 and 7 years. Previous studies in such young children have been under sedation (Funakoshi et al., 2016), during sleep (Fransson et al., 2011; Manning et al., 2013), or included children older than 7 years of age (de Bie et al., 2012; Winkler et al., 2014; Muetzel et al., 2016; Sato et al., 2016). Further, compared to the Dutch cohort studies that mostly included European Caucasian children (de Bie et al., 2012; Muetzel et al., 2016), our study included equal number of children from both African American and Caucasian backgrounds, which is comparable to the captured population of Shelby County, Tennessee, USA.

Importantly, we demonstrate that the primary networks, such as the visual, auditory, sensory, motor, default mode, frontoparietal, executive control, and cerebellar networks are present in healthy 6- and 7-year olds and that they are consistent with those seen in healthy older children and adults. Further, The DMN identified in our study is similar to that reported in a smaller groups of typically developing children aged between 5 and 8 years scanned in awake state (de Bie et al., 2012) and under sedation (Funakoshi et al., 2016). Our findings suggest that the RSNs in a small racially and socioeconomically diverse cohort is representative of the larger pediatric population, and may be sufficient to examine physiological and pathological alterations in these salient RSNs. For example, the monitoring these robust networks thorough childhood can potentially be useful in early detection and follow up of childhood disorders such as autism spectrum disorder, epilepsy, and attention deficit hyperactivity disorder (Mohan et al., 2016). Particularly, we were able to label networks we believe to be the executive control network based off the correlative data with the adult sample. However, compared to the larger cohort study that also included children slightly older than our cohort (Muetzel et al., 2016), the resolution of this network appeared to be weaker in our study. This may be a result of the procedures used for data preprocessing and de-noising, the small sample size, or the young age of participants in our study.

A smaller sample size does have shortcomings when compared to larger sample sizes. A single individual attributes more influence over the group's data, which allows artifact such as noise and movement to influence results more profoundly (Desmond and Glover, 2002). During the cleaning of the data, the noise attributed to movement was typically found at the beginning of each decomposition and explained up to 30% of the variance observed. The removal of movement components that were due to lateral, rotational, or anterior-posterior movements could potentially bias the results, specifically the auditory, temporal, visual, and frontal networks. To reduce the possibility of this error, the cleaning process used spatial and temporal patterns along with the eigenvalues of the decomposition to evaluate the quality of each independent component. Furthermore, the cleaning process was performed at three thresholds for component removal: liberal (20–25% or 12.5 ± 4.0 components removed), moderate (40–60% or 25.2 ± 4.3), and conservative (60–80% or 32.1 ± 7.7). The ICs identified with the moderate threshold revealed optimal cross-correlations coefficients and spatial distribution patterns consistent with the adult brain networks. In case of both liberal and conservative thresholding, the spatial cross correlations were lower and the many networks were found to be less resolved with liberal thresholding and were completely absent, when conservative threshold was used. Our findings are consistent with other reports that show that noise correction beyond a certain threshold is detrimental (Bright and Murphy, 2015). Therefore, we believe that the data presented here are valid and are not adversely influenced by noise correction.

The sample population in our study is young in age, which presents a unique addition of noise to the sample (Thomason et al., 2005). Children at the age of 6- to 7-years are more prone to movement and noncompliance during the scanning procedures. Other studies of young populations have used early stages of sleep scanning (Manning et al., 2013) and mouthpiece/bite-holding apparatus (Thomason et al., 2011) as a means to reduce the inherent movement of the participants during the scan. Both methodologies reported results comparable to what is presented in our findings in terms of discovering the major accepted neural networks, which are believed to be present during gestation (Smyser et al., 2010) or at birth (Fransson et al., 2007). In our study, we had to exclude data from 10 children, which is reasonable at this age. There were no statistically significant differences in demographic measures, such as age, BMI percentile, gestational age, IQ, sex, or race between the individuals who included in the final analysis and those removed from the study. Additionally, there was no statistically significant influence of IQ, demographic measures of gender, race, age and gestational age at birth, or anthropometric parameters of height, weight, and BMI on spatial distribution of RSNs, indicating to the homogeneity of our group. Given the aforementioned difficulties in a young pediatric cohort, the sample size in our study is a realistic aim for other cohorts of similar demographics. The cost and labor to create large studies of certain characteristics is certainly a barrier to further research, but we have shown that major RSNs could be resolved in a small sample size representative of the catchment area.

Further, the areas that varied the most consistently between our population and the Smith data (Smith et al., 2009) were within the frontal lobe and cingulate cortex. Specifically, the frontal regions had fewer significant voxels in the DMN and dorsal attention networks (see Figure 2-IC 1 and IC 4), and the cingulate cortex activity was not present in our data in the motor (IC 8), anterior default mode (IC 15) and the executive control (IC 17) networks. As our participants were still growing, the executive function portion of the brain may not be fully developed, indicating lesser degree of coupling or integration with sensory and motor regions. Other studies have shown that higher cognitive function develops through pre-school age and continues into puberty (Moriguchi and Hiraki, 2013). This is also consistent with the report of age related changes seen in the frontoparietal, DMN and executive control networks as well as increasing connectivity between the frontal and parietal regions (Muetzel et al., 2016). Another notable finding in our study is the separation of somatosensory and motor networks which in adults resolved as one RSN (Muetzel et al., 2016). In our cohort, the IC that overlapped the most with the adult sensorimotor network was labeled as somatosensory as it predominantly included the postcentral gyrus, and inferior parietal lobule. However, this IC also had significant clusters in the precentral gyrus, the SMA, and cerebellum. The IC that was labeled as motor network in our study primarily included precentral gyrus relating to hand and mouth primary motor cortices as well as dorsal and ventral premotor cortex. This motor RSN may represent the developing fine motor and speech networks in children. The sensory and motor networks observed in previous studies in awake children also had similar spatial patterns (de Bie et al., 2012; Muetzel et al., 2016). This observation is consistent with the finding in adults, where the RSN identified as sensorimotor includes pre- and post-central gyrus, premotor cortices, the supplementary motor area (SMA), the cingulate cortex, and parietal association cortices, indicating to an integrated somatosensory and motor system.

The finding of correlative RSNs networks in the primary sensory and motor domains in this group is consistent with the finding that in infancy, the motor, somatosensory, auditory, and visual cortices exhibited strong connectivity to other brain regions (Fransson et al., 2011). This pattern changes over the course of development and in adults, the prefrontal and association cortices demonstrate greater connections to other brain regions (Fransson et al., 2011). Therefore, the prefrontal and association cortices are better visualized in adult RSNs. Future longitudinal studies would be of value to further elucidate the development of these networks and inter-network connections.

In a small, diverse, young pediatric population, we have demonstrated that the visual, auditory, temporal, sensorimotor, frontoparietal, executive control, and DMNs are developed enough to be detected by similar means as the adult and large scale pediatric studies. However, the observation that the present findings are not fully replicated in a symmetrical manner to those of previous adult (Smith et al., 2009) and, the smaller number of children (de Bie et al., 2012), studies, does merit some discussion. For example, these comparative differences may be due to previously mentioned sources of induced noise, as well as neurobiological differences in children and adults (Jolles et al., 2011). Moreover, while the aforementioned RSNs may be stable across individuals, the small sample size of the current study suggests some caution when interpreting the significance of the present findings. Accordingly, addressing this limitation would be necessary in future studies wishing to determine the degree to which RSNs in 6–7 year olds with neurodevelopmental disorders or neuronal injury may deviate from the normative profile, and determining the utility of resting-state fMRI as a diagnostic tool in clinical pediatric populations.

## Conclusion

We have shown that the ICA approach to fMRI analysis is a robust technique that can replicate the results of larger studies in adults and children in a smaller sample study of awake 6- to 7-year-old children. As our understanding of cognitive changes shown by RSNs increases, the utility of the ICA approach toward diagnosing conditions with complex diagnoses will increase alongside. The data shown here could serve as a diverse normative dataset to compare 6- to 7-year-old patients with suspected neuropsychiatric disorder against a homogenous, baseline sample set. Future studies investigating the longitudinal growth and changes in RSNs as measured by ICA-based resting-state fMRI analyses in diverse pediatric population will provide further insight on the development of the intrinsic connectivity networks. The MRI data and the derived RSNs from this study will be made available to other researchers via the CANDLE Study Data Repository (www.candlestudy.com).

## Author contributions

All authors had full access to all the data in the study and take responsibility for the integrity of the data and the accuracy of the data analysis. Study concept and design: EV, SN, RR, AP. Data Acquisition: BB, CT, FT, AC, RR, EV, SN. Data Analysis and interpretation: BB, CT, FT, AC, AP, RR, EV, SN. Drafting of the manuscript: CT, SN, RR, EV. Contributing important intellectual content in manuscript review: CT, BB, SN, RR, EV, AP. Obtained funding: EV. Study supervision: EV.

## Funding

The study was funded by the Children's Foundation Research Institute, Le Bonheur Children's Hospital, Memphis, TN, USA (PI: EV). CT was funded by the the University of Tennessee Health Science Center NIH Medical School Research Fellowship Program.

### Conflict of interest statement

The authors declare that the research was conducted in the absence of any commercial or financial relationships that could be construed as a potential conflict of interest.
